# Cuprizone feed formulation influences the extent of demyelinating disease pathology

**DOI:** 10.1038/s41598-021-01963-3

**Published:** 2021-11-19

**Authors:** Lillian M. Toomey, Melissa Papini, Brittney Lins, Alexander J. Wright, Andrew Warnock, Terence McGonigle, Sarah C. Hellewell, Carole A. Bartlett, Chidozie Anyaegbu, Melinda Fitzgerald

**Affiliations:** 1 Curtin Health Innovation Research Institute, Curtin University, Bentley, WA 6102 Australia; 2 Perron Institute for Neurological and Translational Science, Sarich Neuroscience Research Institute Building, 8 Verdun St, Nedlands, WA 6009 Australia

**Keywords:** Multiple sclerosis, Myelin biology and repair

## Abstract

Cuprizone is a copper-chelating agent that induces pathology similar to that within some multiple sclerosis (MS) lesions. The reliability and reproducibility of cuprizone for inducing demyelinating disease pathology depends on the animals ingesting consistent doses of cuprizone. Cuprizone-containing pelleted feed is a convenient way of delivering cuprizone, but the efficacy of these pellets at inducing demyelination has been questioned. This study compared the degree of demyelinating disease pathology between mice fed cuprizone delivered in pellets to mice fed a powdered cuprizone formulation at an early 3 week demyelinating timepoint. Within rostral corpus callosum, cuprizone pellets were more effective than cuprizone powder at increasing astrogliosis, microglial activation, DNA damage, and decreasing the density of mature oligodendrocytes. However, cuprizone powder demonstrated greater protein nitration relative to controls. Furthermore, mice fed control powder had significantly fewer mature oligodendrocytes than those fed control pellets. In caudal corpus callosum, cuprizone pellets performed better than cuprizone powder relative to controls at increasing astrogliosis, microglial activation, protein nitration, DNA damage, tissue swelling, and reducing the density of mature oligodendrocytes. Importantly, only cuprizone pellets induced detectable demyelination compared to controls. The two feeds had similar effects on oligodendrocyte precursor cell (OPC) dynamics. Taken together, these data suggest that demyelinating disease pathology is modelled more effectively with cuprizone pellets than powder at 3 weeks. Combined with the added convenience, cuprizone pellets are a suitable choice for inducing early demyelinating disease pathology.

## Introduction

The cuprizone-induced animal model is a toxin-based method of producing demyelinating disease similar to that observed in the brains of people with MS. Cuprizone (or *bis*-cyclohexanone oxaldihydrazone) is a copper chelating agent that is orally administered to rodents to induce oligodendroglial death, glial cell activation and demyelination via mitochondrial dysfunction^1–3^. Cuprizone induces mitochondrial injury and oxidative stress by increasing mitochondrial production of superoxide anions^4^, reducing copper-dependent cytochrome *c* oxidase and monoamine oxidase activity^1^, decreasing copper-zinc superoxide dismutase^5^ and glutathione^6^ antioxidant activity, and inhibiting electron transport chain complex IV^7^. Oligodendrocytes are particularly vulnerable to cuprizone-induced mitochondrial dysfunction^7^, resulting in apoptosis^1^ and subsequent loss of myelin^4^. In addition, mitochondrial dysfunction in oligodendrocytes results in endoplasmic reticulum stress^8–10^, causing further downregulation of myelin proteins^11^ and reduced myelin lipid synthesis^4^. In humans, copper deficiency results in CNS demyelination^12–14^. Furthermore, mitochondrial electron transport chain complex IV dysfunction in oligodendrocytes has been found in type III active MS lesions^15^. However, cuprizone may also induce its biological effects via copper-independent mechanisms^16^. Concomitant copper supplementation alongside cuprizone administration does not completely attenuate the observed degenerative effects^17^. Furthermore, it has been suggested that the effects of cuprizone on oligodendrocyte metabolism are related to a Schiff base formation binding with the amino acid metabolism coenzyme pyridoxal 5′-phosphate, and completely independent of copper chelation^18^. A recent study has also discovered that the copper chelating action of cuprizone produces dysregulation of iron homeostasis, leading to ferroptosis-mediated loss of oligodendrocytes and myelin^19^.

Irrespective of its mechanism of action, cuprizone is a widely utilised and validated toxin for modelling CNS demyelination that is not directly associated with peripheral autoimmunity^20^. Furthermore, cuprizone intoxication is a useful tool for studying the processes of remyelination, as withdrawal of cuprizone typically results in remyelination in the CNS of the intoxicated animal^21^, albeit at a significantly slower rate following chronic cuprizone intoxication^22^.

While cuprizone mainly affects oligodendrocyte myelination, indirect effects on other cell types in the CNS can be observed. For example, as early as 1 week into cuprizone intoxication, microglia are activated within both the cortex and the corpus callosum^23^. Astrocytes exhibit morphological changes as early as 1 week, with numbers of astrocytes significantly increasing from 3 weeks of cuprizone administration^24^. Also by 3 weeks, OPCs have accumulated in the corpus callosum, particularly in the caudal regions, ready to act as a progenitor pool for the differentiation and maturation of new oligodendrocytes^25,26^.

The reliability and reproducibility of a cuprizone-induced model of demyelinating disease depends critically on the animals ingesting the full dose of cuprizone consistently in different studies. Traditionally, cuprizone has been delivered in powdered form mixed into ground rodent chow, but in recent years pelleted formulations of cuprizone that allow more convenient handling and feeding have become available. Despite some evidence suggesting that pelleted formulations of cuprizone can be less effective^27,28^, the use of cuprizone in a pelleted form is growing in popularity, with many research groups successfully utilising the pelleted form at chronic timepoints^29–36^. However, one study in particular noted that their cuprizone-containing pellets failed to induce demyelination despite toxicological analysis of the pellets confirming that they still contained active cuprizone^28^. Another study has directly compared the potency of cuprizone pellets and powder and demonstrated that cuprizone powder was more effective at inducing demyelinating pathology than pellets at an equivalent dose of cuprizone^27^. This study used a 0.25% dose of cuprizone, rather than the more commonly used 0.2% cuprizone dose^24^ and included 2 weeks remyelination after the 3 week cuprizone administration^27^. Effects of the different feed formulations on oligodendroglial death and astrogliosis were not assessed. Information on the effects of pelleted cuprizone at the early 3 week timepoint during demyelination is lacking. In the present study, we assessed the potency of pelleted and powdered cuprizone at an early demyelinating timepoint. 0.2% cuprizone was delivered in both powdered and pelleted form and outcomes were assessed following 3 weeks of cuprizone administration. Various cytochemical and immunohistochemical parameters, including oligodendroglial death, gliosis, oxidative stress and demyelination, were investigated to determine the extent to which powdered and pelleted cuprizone differ in their capacity to induce the hallmarks of cuprizone-intoxication in the CNS.

## Results

### Effects of cuprizone feed formulation on body weight

**Figure 1:**
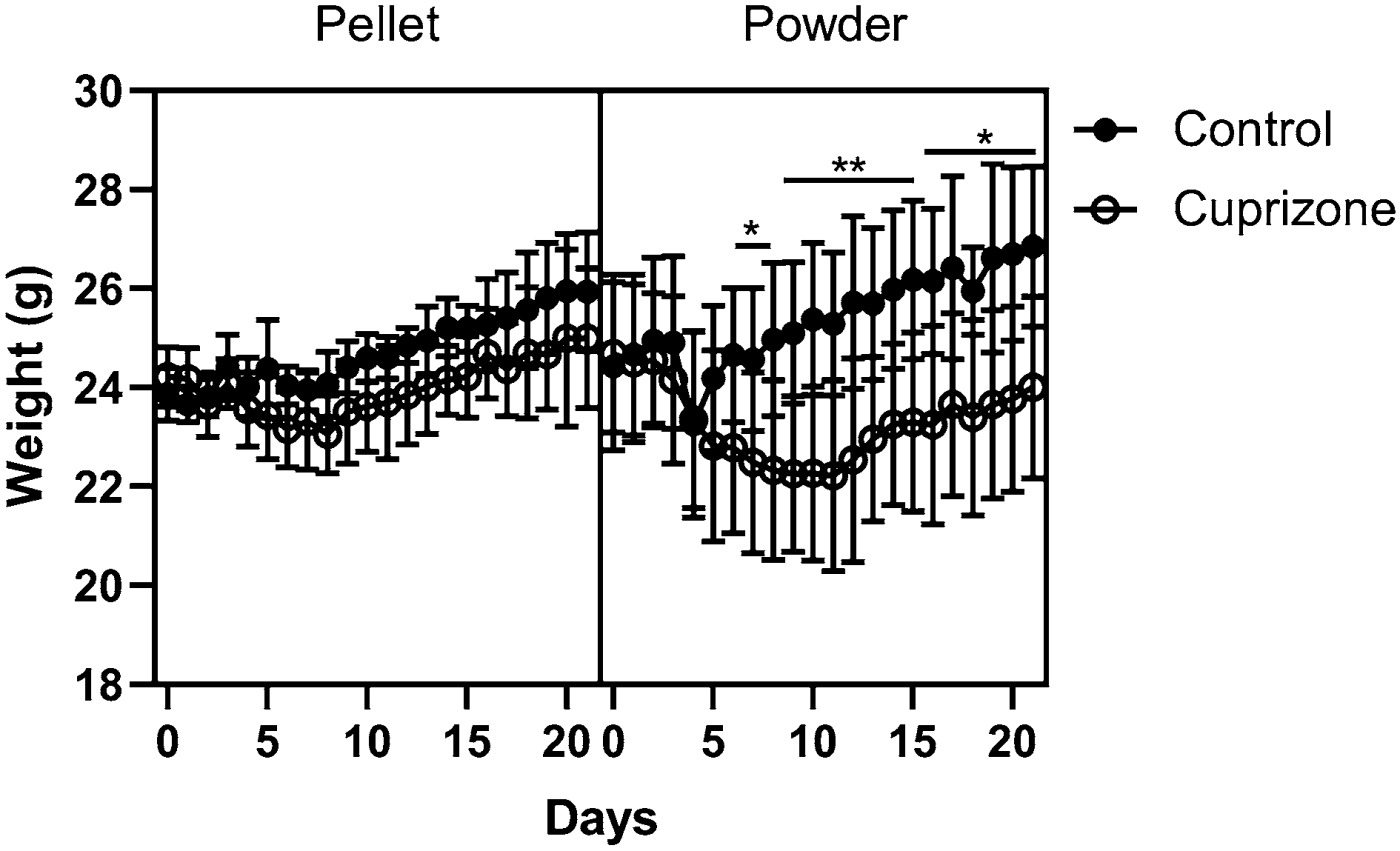
Effects of cuprizone feed formulation on animal weights. Animals were weighed daily to assess weight loss throughout cuprizone administration. Cuprizone was delivered in either pelleted or powdered form. N = 6 mice per group; graph displays mean weight ± SEM. Significant differences are indicated by *p ≤ 0.05, **p ≤ 0.01.

Following 3 weeks on diets of control powder, cuprizone-containing powder, control pellet or cuprizone-containing pellet, mice were assessed for weight loss, a widely recognised hallmark of cuprizone intoxication^34^. Post-hoc analyses revealed that mice administered cuprizone powder had significantly lower body weight than those fed control powder from 7 days of cuprizone until the end of the study (p < 0.05; Fig. 1). However, mice fed cuprizone pellets did not have any significant differences in body weight compared to their control pellet counterparts at any timepoint (p > 0.05). There were no differences between cuprizone pellet and cuprizone powder (p > 0.05).

### Effects of cuprizone feed formulation on inflammatory responses

**Figure 2:**
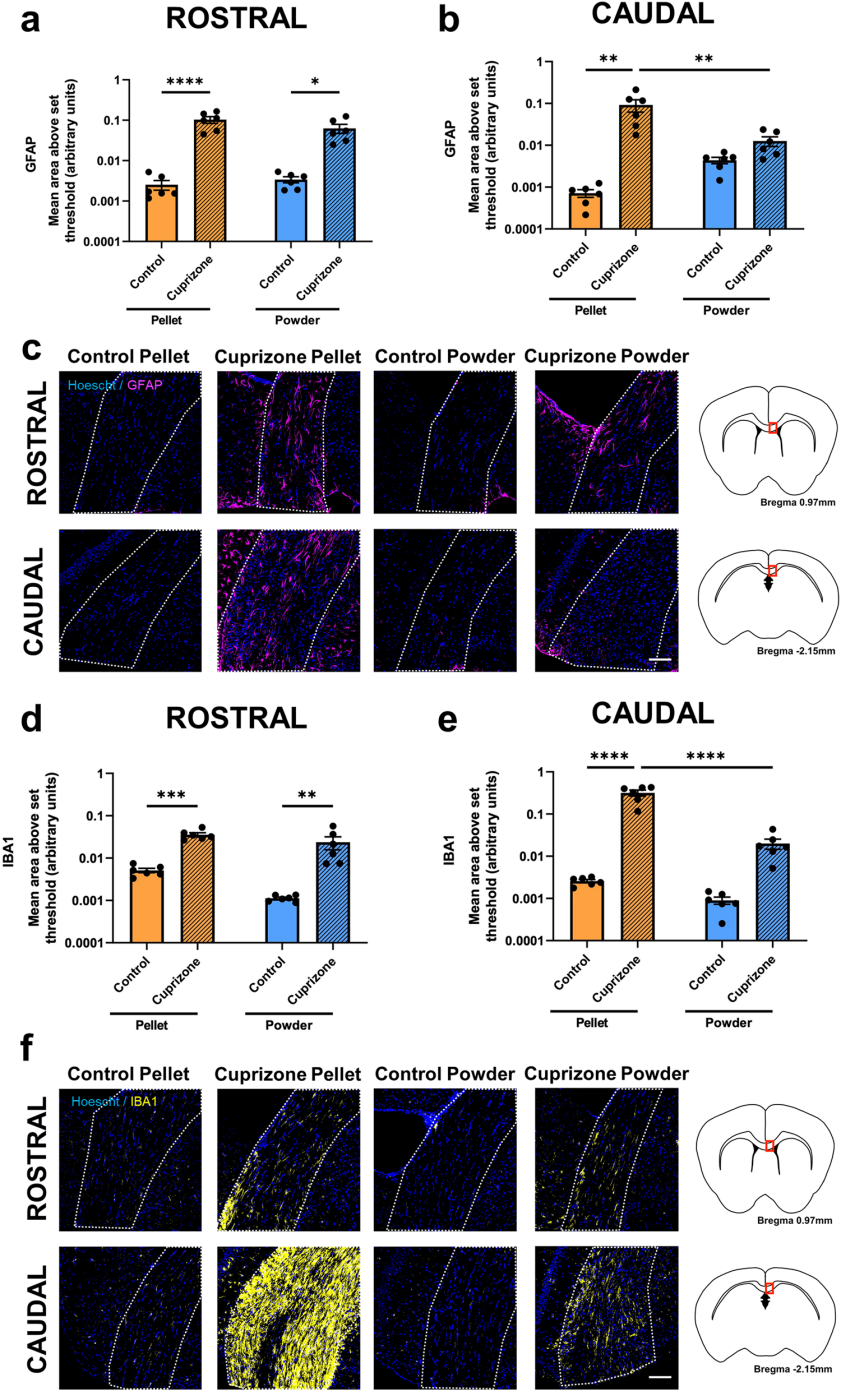
Effects of cuprizone feed formulation on glial reactivity. Area of immunointensity of GFAP in the rostral (**a**) and caudal (**b**) corpus callosum was assessed to determine the level of astrocyte reactivity. Area of immunointensity of IBA1 in the rostral (**d**) and caudal (**e**) corpus callosum was assessed to determine the level of microglial activation. N = 6 mice per group, graphs display individual data points overlayed on a bar displaying the mean ± SEM. Significant differences are indicated by *p ≤ 0.05, **p ≤ 0.01, ***p ≤ 0.001, ****p ≤ 0.0001. Representative images of the area of GFAP (**c**) and IBA1 (**f**) immunoreactivity is shown, scale bars = 100 µm. Area of the corpus callosum analysed is denoted by dotted lines. This area is indicated by the red box in the coronal overviews illustrating the rostral and caudal regions of the corpus callosum (**c**,**f**). The stereotaxic coordinates are indicated.

Within the rostral corpus callosum the area of GFAP immunoreactivity, indicative of astrocyte reactivity, significantly increased when cuprizone was delivered in the form of pellets compared to control pelleted feed (p < 0.0001; Fig. 2a,c). Similarly, the area of GFAP immunoreactivity increased with powdered cuprizone delivery when compared to control powdered feed (p = 0.0158). There were no significant differences between control groups or cuprizone groups (p = 0.999 and p = 0.144 respectively). Within the caudal corpus callosum, mice fed pelleted cuprizone again had a significantly greater area of GFAP immunoreactivity than those fed control pellets (p = 0.002; Fig. 2b,c). However, in the caudal region of the corpus callosum, animals fed cuprizone in the form of powder did not have increased GFAP immunoreactivity when compared to those fed control powder (p = 0.980). When cuprizone was delivered in the form of pellets, there was a significantly greater area of GFAP immunoreactivity than when it was delivered in the form of powder (p = 0.007). There were no differences between the two control groups (p = 0.998).

Within the rostral corpus callosum, the area of IBA1 immunoreactivity, indicative of microglial activation, significantly increased when cuprizone was delivered in the form of pellets compared to control pelleted feed (p = 0.0005; Fig. 2d,f). There was also an increase in the area of IBA1 immunoreactivity when cuprizone was delivered as powder compared to control powdered feed (p = 0.0095). There were no differences between the two control groups (p = 0.920) or the two cuprizone groups (p = 0.254). Within the caudal corpus callosum, mice fed pelleted cuprizone had a significantly greater area of IBA1 immunoreactivity than those fed control pellets (p < 0.0001; Fig. 2e,f). It is worth noting that IBA1 immunoreactivity in these cuprizone pellet fed animals was considerably more pronounced in caudal than rostral corpus callosum (Fig. 2f). However in caudal corpus callosum there was no significant increase in the area of IBA1 immunoreactivity when cuprizone was delivered as powder compared to control powdered feed (p = 0.9527). Similarly to the findings with GFAP, when cuprizone was delivered in the form of pellets, there was significantly more IBA1 immunoreactivity than when it was delivered in the form of powder (p < 0.0001). There were no differences between the two control groups (p > 0.999).

### Effects of cuprizone feed formulation on oxidative stress

**Figure 3:**
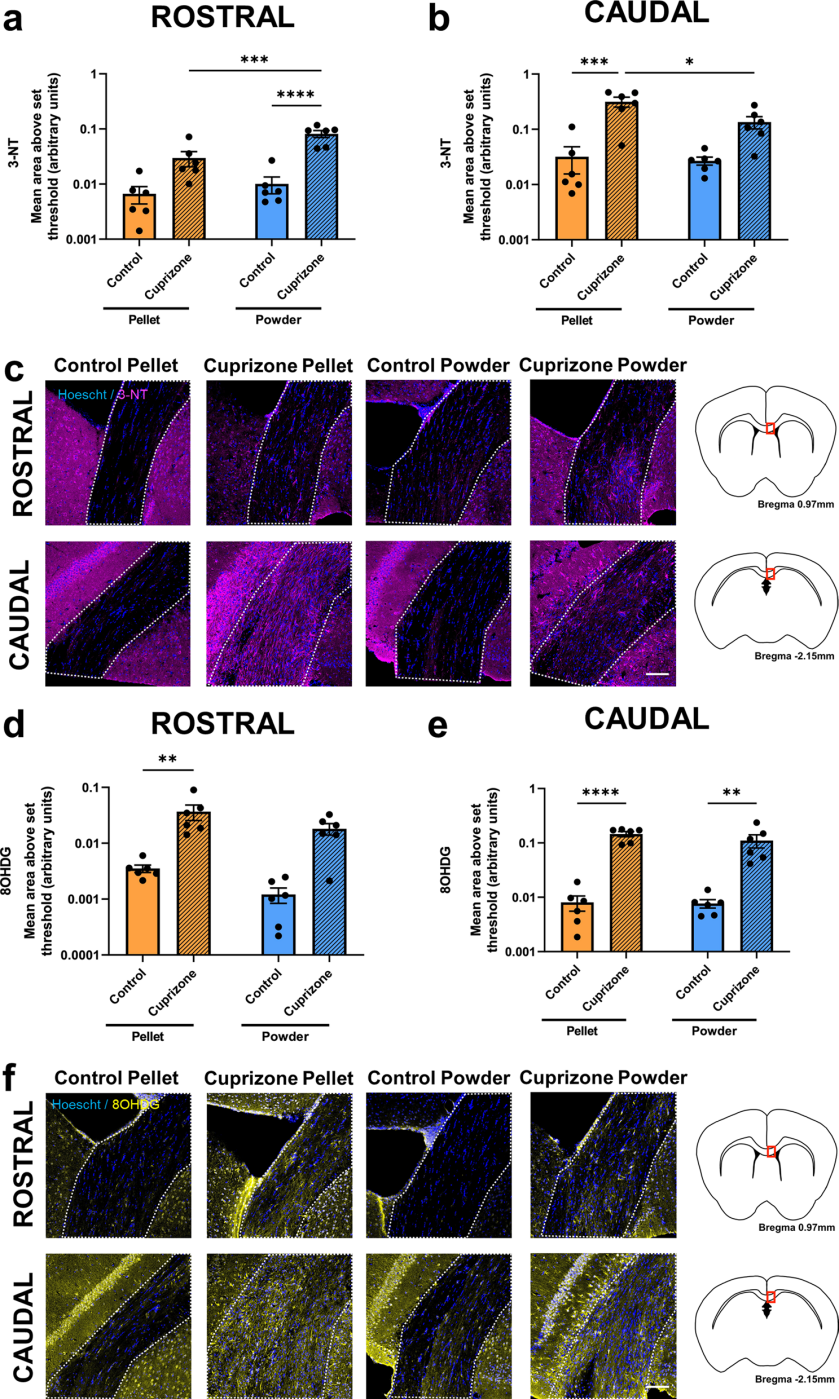
Effects of cuprizone feed formulation on oxidative stress. Area of immunointensity of 3-NT in the rostral (**a**) and caudal (**b**) corpus callosum was assessed to determine the level of protein nitration. Area of immunointensity of 8OHDG in the rostral (**d**) and caudal (**e**) corpus callosum was assessed to determine the level of oxidative DNA damage. N = 6 mice per group, graphs display individual data points overlayed on a bar displaying the mean ± SEM. Significant differences are indicated by *p ≤ 0.05, **p ≤ 0.01, ***p ≤ 0.001, ****p ≤ 0.0001. Representative images of the area of 3-NT (**c**) and 8OHDG (**f**) immunoreactivity are shown, scale bars = 100 µm. Area of the corpus callosum analysed is denoted by dotted lines. This area is indicated by the red box in the coronal overviews illustrating the rostral and caudal regions of the corpus callosum (**c**,**f**). The stereotaxic coordinates are indicated.

Within the rostral corpus callosum the area of 3-NT immunoreactivity, indicative of protein nitration, was not affected by delivery of cuprizone in the form of pellets (p = 0.1929, Fig. 3a,c). In contrast, the area of 3-NT significantly increased when cuprizone was delivered in the form of powder compared to control powdered feed (p < 0.0001). When cuprizone was delivered in the form of powder, there was a significantly greater area of 3-NT immunoreactivity than when it was delivered in the form of pellets (p = 0.0008). There were no differences between the two control groups (p = 0.9897). In contrast, within the caudal corpus callosum, mice fed pelleted cuprizone had a significantly greater area of 3-NT immunoreactivity than those fed control pellets (p = 0.0002; Fig. 3b,c). Similarly to the pattern of IBA1 immunoreactivity, 3-NT immunoreactivity was more pronounced caudally than rostrally (Fig. 3c). However there was no increase in the area of 3-NT immunoreactivity when cuprizone was delivered as powder compared to control powdered feed (p = 0.2051). When cuprizone was delivered in the form of pellets, there was a significantly greater area of 3-NT immunoreactivity than when it was delivered in the form of powder (p = 0.0138). There were no differences between the two control groups (p = 0.9997).

Within the rostral corpus callosum the area of 8OHDG immunoreactivity, indicative of oxidative DNA damage, significantly increased when cuprizone was delivered in the form of pellet compared to control pelleted feed (p = 0.005; Fig. 3d,f). However there was no significant difference when cuprizone was delivered as powder, compared to control powdered feed (p = 0.2353). There were no differences between the two control groups (p = 0.9931) or the two cuprizone groups (p = 0.1679). Within the caudal corpus callosum, mice fed pelleted cuprizone had a significantly greater area of 8OHDG immunoreactivity than those fed control pellets (p < 0.0001; Fig. 3e,f). There was also an increase in the area of 8OHDG immunoreactivity when cuprizone was delivered as powder compared to control powdered feed (p = 0.0021). There were no differences between the two control groups (p > 0.9999) or the two cuprizone groups (p = 0.487).

### Effects of cuprizone feed formulation on tissue swelling

**Figure 4:**
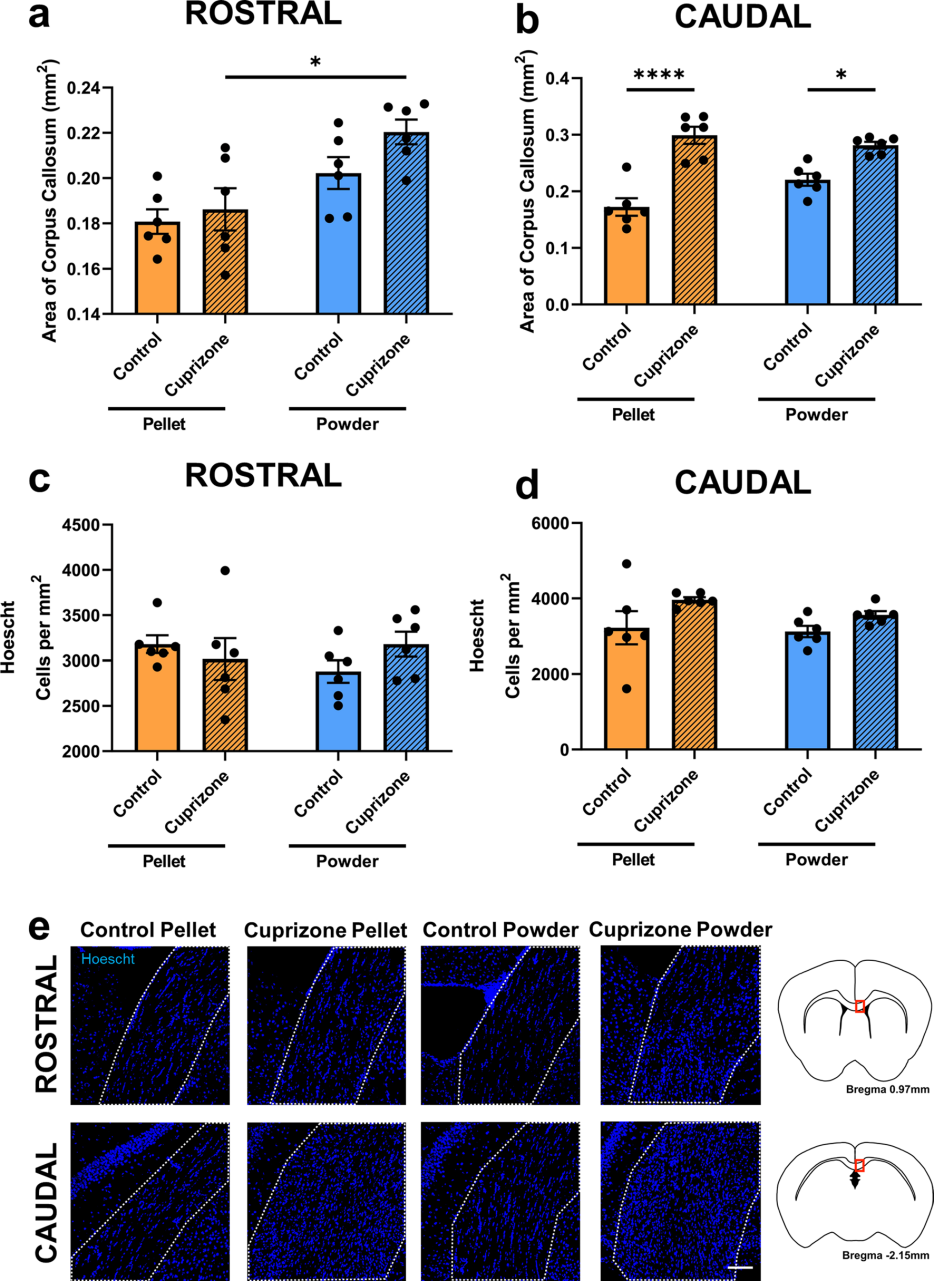
Effects of cuprizone feed formulation on tissue swelling. The area of the corpus callosum in the rostral (**a**) and caudal (**b**) areas was assessed. The density of Hoechst+ cells was also quantified in the rostral (**c**) and caudal (**d**) corpus callosum. N = 6 mice per group, graphs display individual data points overlayed on a bar displaying the mean ± SEM. Significant differences are indicated by *p ≤ 0.05, **p ≤ 0.01, ***p ≤ 0.001, ****p ≤ 0.0001. Representative images Hoechst+ cells (**e**) is shown, scale bars = 100 µm. Area of the corpus callosum analysed is denoted by dotted lines. This area is indicated by the red box in the coronal overviews illustrating the rostral and caudal regions of the corpus callosum (**e**). The stereotaxic coordinates are indicated.

The area of the rostral corpus callosum was not significantly different between either of the two pellet groups (p = 0.9462; Fig. 4a,e) or powder groups (p = 0.2868). There was also no difference between the two control groups (p = 0.1659), but there was a significant increase in the size of the rostral corpus callosum when cuprizone was delivered as a powder compared to pellets (p = 0.0122). The area of the caudal corpus callosum was significantly larger in mice fed pelleted cuprizone than those fed control pellets (p < 0.0001; Fig. 4b,e). There was also an increase in the area of the caudal corpus callosum when cuprizone was delivered as powder compared to control powdered feed (p = 0.0114). There were no differences between the two control groups (p = 0.0552) or the two cuprizone groups (p = 0.7506).

To determine whether the enlarged corpus callosum was due to a general increase in cell density, the number of Hoechst+ cells per mm^2^ was quantified. In the rostral (p > 0.05; Fig. 4c,e) and caudal (p > 0.05, Fig. 4d,e) corpus callosum there were no significant differences between groups. This suggests that variation in size of the corpus callosum is due to swelling, rather than increased cell density.

### Effects of cuprizone feed formulation on oligodendroglia

**Figure 5:**
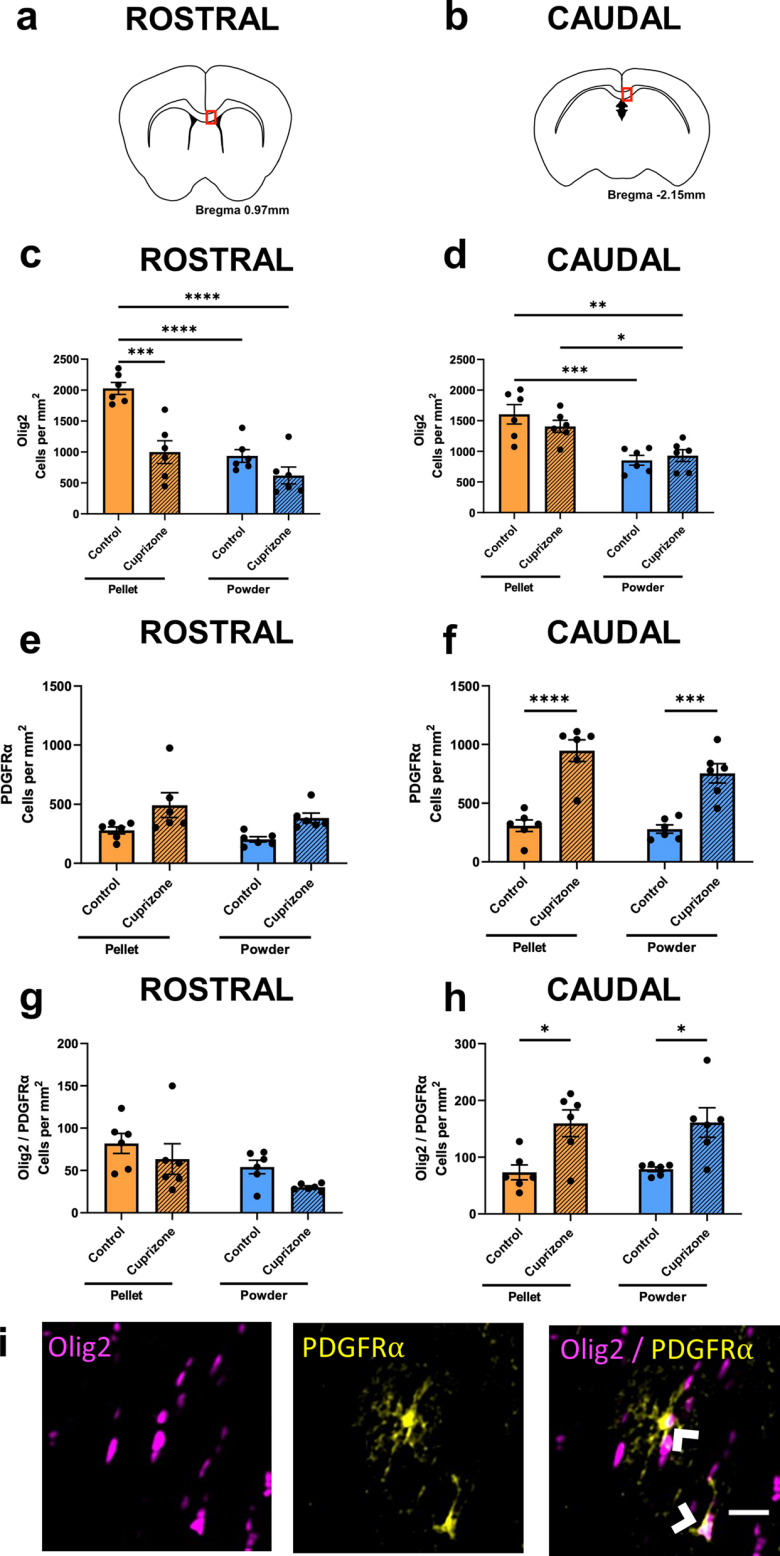
Effects of cuprizone feed formulation on densities of Olig2+, PDGFRα+ and Olig2+/PDGFRα+ cells. The area of the rostral and caudal corpus callosum analysed is denoted by the red box in the coronal illustrations of the brain (**a**,**b**). The density of Olig2+ cells (**c**,**d**), PDGFRα+ cells (**e**,**f**) and Olig2+/PDGFRα+ cells (**g**,**h**) were quantified in the rostral and caudal corpus callosum. N = 6 mice per group, graphs display individual data points overlayed on a bar displaying the mean ± SEM. Significant differences are indicated by *p ≤ 0.05, **p ≤ 0.01, ***p ≤ 0.001, ****p ≤ 0.0001. (**i**) Representative images of Olig2+, PDGFRα+ and Olig2+/PDGFRα+ cells, indicated with arrow heads; scale bar = 25 µm.

Within the rostral corpus callosum the density of Olig2+ cells, broadly indicative of oligodendroglia, significantly decreased when cuprizone was delivered in the form of pellets compared to control pelleted feed (p = 0.0001; Fig. 5a,c,i). In contrast, cuprizone powder did not result in a significantly decreased density of Olig2+ cells, when compared to mice fed control powder (p = 0.3723). Interestingly, however, when powdered cuprizone was compared to pelleted control, the density of Olig2+ cells decreased in the caudal (p < 0.0021) and rostral (p < 0.0001) corpus callosum (Fig. 5a–d,i). When the control feed was delivered as a powder rather than as pellets, there was a significant decrease in the density of Olig2+ cells (p < 0.0001). There was no significant difference between the two cuprizone groups (p = 0.2262). Within the caudal corpus callosum, there were no differences between mice fed control pellets and cuprizone pellets (p = 0.6126; Fig. 5b,d,i), or those fed control powder and cuprizone powder (p = 0.9609). Again, control powder led to lower density of Olig2+ cells than in mice fed control pellets (p = 0.0007), cuprizone powder also resulted in a lower density of Olig2+ cells than cuprizone pellet (p = 0.0332).

To quantify OPCs, PDGFRα was utilised both alone, and in combination with Olig2^37^. Within the rostral corpus callosum there were no significant differences in the density of PDGFRα+ cells between any of the groups (p > 0.05; Fig. 5a,e,i). Within the caudal corpus callosum, the number of PDGFRα+ cells significantly increased when cuprizone was delivered as pellets compared to control pelleted feed (p < 0.0001; Fig. 5b,f,i). Similarly, the number of PDGFRα+ cells significantly increased when cuprizone was delivered in the form of powder compared to control powdered feed (p = 0.0005). There was no difference between the two control groups (p = 0.9908) or the two cuprizone groups (p = 0.2258).

Similar to the analyses of PDGFRα+ cells, there were no differences in density of Olig2+/PDGFRα+ cells between any of the groups in rostral corpus callosum (p > 0.05; Fig. 5a,g,i). Within the caudal corpus callosum, the density of Olig2+/PDGFRα+ cells significantly increased when cuprizone was delivered as pellets compared to control pelleted feed (p = 0.0195; Fig. 5b,h,i). Similarly, the number of Olig2+/PDGFRα+ cells significantly increased when cuprizone was delivered in the form of powder compared to control powdered feed (p = 0.0272). There was no difference between the two control groups (p = 0.9969) or the two cuprizone groups (p > 0.9999).

**Figure 6:**
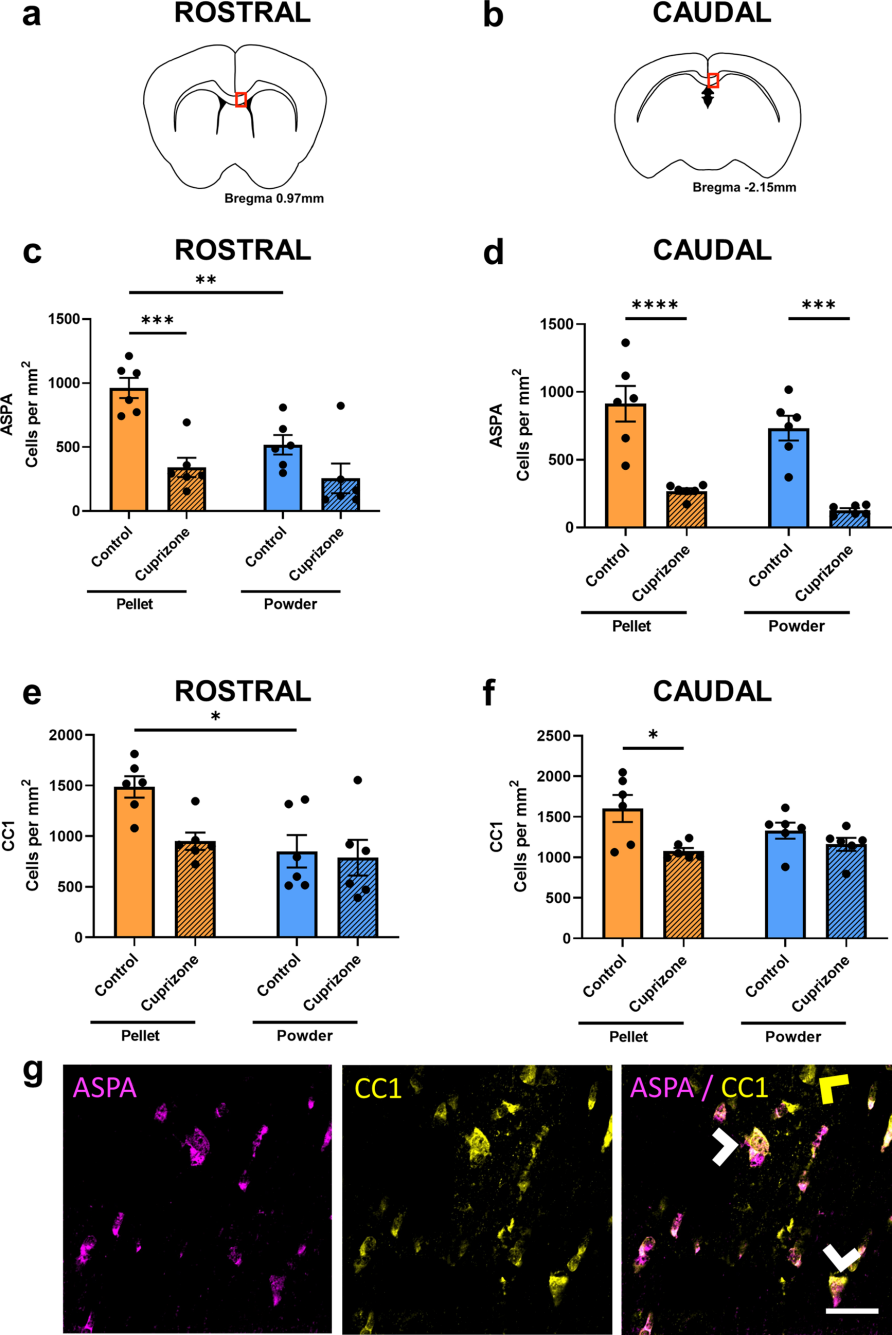
Effects of cuprizone feed formulation on densities of ASPA+ and CC1+ cells. The area of the rostral and caudal corpus callosum analysed is denoted by the red box in the coronal illustrations of the brain (**a**,**b**). The density of ASPA+ cells (**c**,**d**) and CC1+ cells (**e**,**f**) were quantified in the rostral and caudal corpus callosum. N = 6 mice per group, graphs display individual data points overlayed on a bar displaying the mean ± SEM. Significant differences are indicated by *p ≤ 0.05, **p ≤ 0.01, ***p ≤ 0.001, ****p ≤ 0.0001. (**g**) Representative images of ASPA+ and CC1+ cells, indicated with white arrow heads; example ASPA-/CC1+ cell indicated with yellow arrow head; scale bar = 25 µm.

ASPA and CC1 were used to enumerate mature oligodendrocytes^38^. Within the rostral corpus callosum there was a significant decrease in the density of ASPA+ cells when cuprizone was delivered as pellets compared to control pelleted feed (p = 0.0004, Fig. 6a,c,g). However, there was no difference in ASPA+ cell density between mice fed control powder or cuprizone powder (p = 0.1851). Interestingly, similar to the Olig2+ cell counts, there was a decrease in the density of ASPA+ cells in the control powder group compared to the control pelleted group (p = 0.0100). There were no significant differences between the two cuprizone groups (p = 0.9017). Within the caudal corpus callosum, there was a significant decrease in the density of ASPA+ cells with both cuprizone pellets (p < 0.0001, Fig. 6b,d,g) and cuprizone powder (p = 0.0002) compared to their relative control groups. There were no differences between control groups (p = 0.4154) or the two cuprizone groups (p = 0.6206).

Within the rostral corpus callosum there was no difference in CC1+ cell density between mice fed cuprizone pellet (p = 0.0536, Fig. 6a,e,g) or cuprizone powder (p = 0.9875) compared to their relative control groups. However, again there was a significant decrease in the density of CC1+ cells in mice fed control powder compared to those fed control pellets (p = 0.0182). There were no differences between cuprizone groups (p = 0.8352). Within the caudal corpus callosum, there was a significant decrease in density of CC1+ cells only when cuprizone was delivered as pellets (p = 0.0114, Fig. 6b,f,g), and not when it was delivered as a powder (p = 0.6875), compared to the relative control groups. There were no significant differences between control groups (p = 0.2943) or cuprizone groups (p = 0.9423). It is worth noting that the density of CC1+ cells was generally higher than the density of ASPA+ cells (Fig. 6a–d) and not all CC1+ cells were ASPA+ (Fig. 6g; yellow arrow).

### Effects of cuprizone feed formulation on myelination

**Figure 7:**
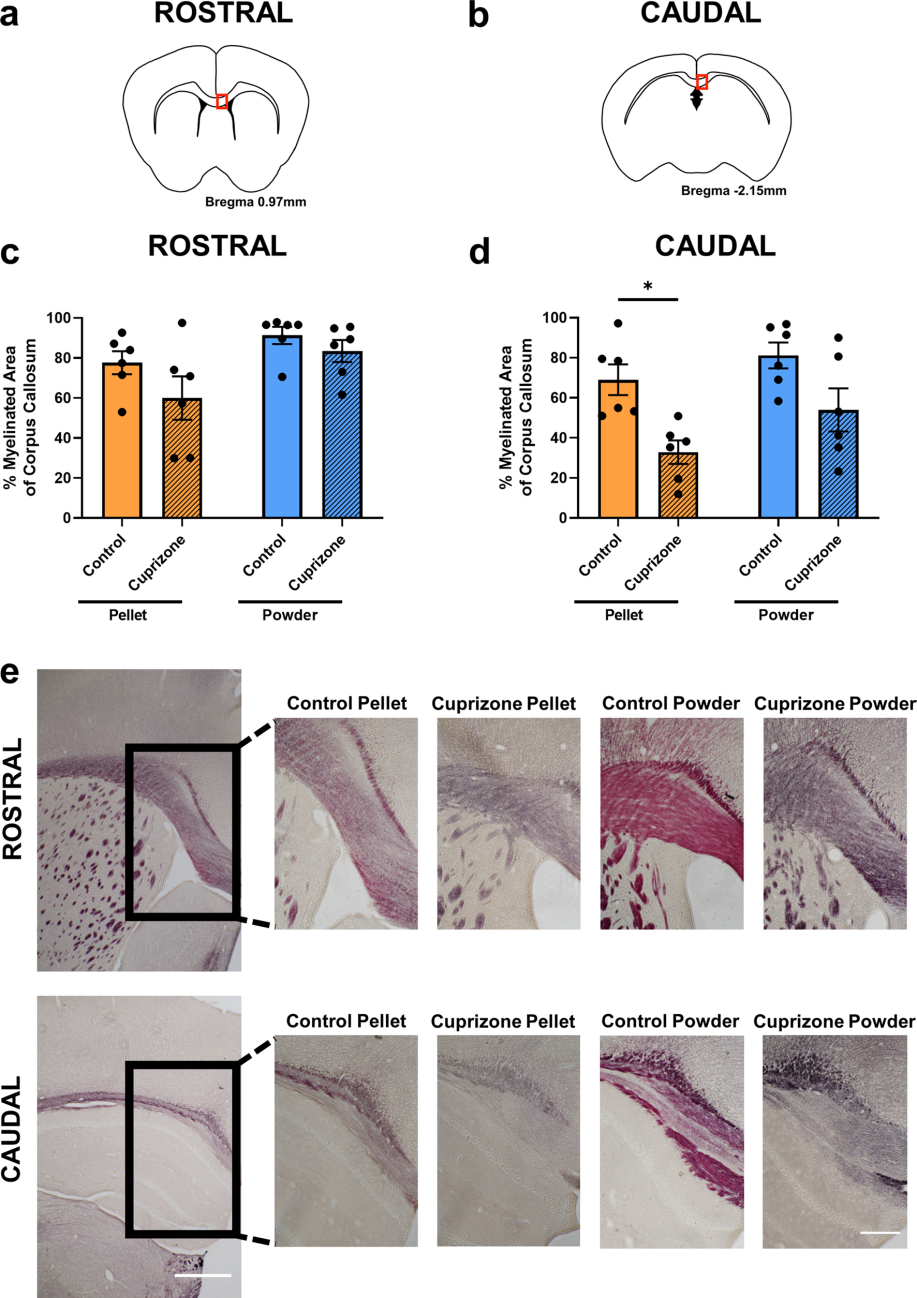
Effects of cuprizone feed formulation on demyelination. The area of the rostral and caudal corpus callosum analysed is denoted by the red box in the coronal illustrations of the brain (**a**,**b**). Percentage area of staining of Black-Gold II in the rostral (**c**) and caudal (**d**) corpus callosum was assessed to determine the level of myelination. N = 6 mice per group, graphs display individual data points overlayed on a bar displaying the mean ± SEM. Significant differences are indicated by *p ≤ 0.05, **p ≤ 0.01, ***p ≤ 0.001, ****p ≤ 0.0001. (**e**) Representative images of the area of Black-Gold II for each group is shown, 4 × images scale bar = 500 µm; 10 × images scale bar = 200 µm.

Utilising the histological stain Myelin Black-Gold II as a marker for myelination, there were no significant differences in the level of myelination within the rostral corpus callosum between groups (p > 0.05, Fig. 7a,c,e). Within the caudal corpus callosum, there was a significant decrease in myelination only when cuprizone was delivered in the form of pellets (p = 0.0199, Fig. 7b,d,e), and not when it was delivered as a powder (p = 0.1025), compared to the relative control groups. There were no significant differences between control groups (p = 0.7038) or cuprizone groups (p = 0.2659). Note, the pink hue present in the control powder group was replicated across multiple staining runs and does not affect the analysis.

## Discussion

Though the efficacy of cuprizone pellets has been debated^27,28^, research has proven that at more chronic timepoints cuprizone pellets can induce reliable demyelination^29–35^. Therefore, in this study we investigated the extent that cuprizone pathology is dependent on feed formulation at an early 3 week timepoint. Across most of the outcome measures analysed, cuprizone pellets were generally better than cuprizone powder at inducing early demyelinating disease pathology compared to their relative controls, particularly in the caudal corpus callosum (Table 1). Cuprizone pellets are a more convenient route of cuprizone administration, as they can be commercially custom made and do not require daily replacement to ensure activity is not compromised by rodent urine. Cuprizone pellets also pose a lower inhalation risk to the researcher than powdered cuprizone.

**Table 1:** Summary of immunohistochemical and histological outcomes.

Outcome measure	Cuprizone pellet relative to control pellet		Cuprizone powder relative to control powder		Cuprizone pellet relative to cuprizone powder		Control powder relative to control pellet	
	Rostral	Caudal	Rostral	Caudal	Rostral	Caudal	Rostral	Caudal
Astrocyte reactivity	↑↑	↑	↑	–	–	↑	–	–
Microglial activation	↑↑	↑↑	↑	–	–	↑↑	–	–
Protein nitration	–	↑↑	↑↑	–	↓↓	↑	–	–
DNA damage	↑	↑↑	–	↑	–	–	–	–
Area of corpus callosum	↓	↑↑	–	↑	–	–	–	–
Hoechst+ cells	–	–	–	–	–	–	–	–
Olig2+ cells	↓↓	–	–	–	–	↑	↓↓	↓↓
PDGFRα+ cells	–	↑↑	–	↑↑	–	–	–	–
Olig2+/PDGFRα+ cells	–	↑	–	↑	–	–	–	–
ASPA+ cells	↓↓	↓↓	–	↓↓	–	–	↓	–
CC1+ cells	–	↓	–	–	–	–	↓	–
Myelination	–	↓	–	–	–	–	–	–

Arrows ↑ and ↓ represent a statistically significant increase or decrease and – represents no significant difference. Single arrows represent p values of greater than 0.001 and less than 0.05, whilst double arrows represent p values ≤ 0.001.

In this study, mice were given equivalent quantities of cuprizone regardless of feed formulation, and feed was replenished in line with standard protocols^34,39^, therefore it is likely that the observed differences between formulations is due to differences in consumption of the feed between different formulations. Weight loss in cuprizone-fed animals over the first week of administration is thought to be indicative that the drug is efficacious at producing demyelinating pathology^34,40^. However, the more pronounced differences in astrocyte reactivity, microglial activation, oxidative stress, tissue swelling and demyelination within the cuprizone pellet group compared to their controls at 3 weeks, despite no significant weight loss, suggest that weight loss may not be a reliable proxy of the extent of demyelinating disease. Instead, the initial weight loss observed in mice fed the cuprizone powder despite reduced cuprizone pathology suggests that the weight loss was due to the mice not ingesting the powdered cuprizone feed. It is plausible that the addition of cuprizone to powdered chow changes the palatability of the feed^41^, but why this did not occur with the cuprizone pellets is currently unclear. Nevertheless, the current data suggest that reduced palatability and subsequent aversion to the powdered cuprizone by the mice may result in the animals initially not ingesting the powdered feed and thus having a reduced or perhaps a delayed effect of cuprizone at the 3 week timepoint. However, a time course comparison study would be needed to fully elucidate whether the reduced ingestion of the feed resulted in a delayed onset of cuprizone-induced pathology. Importantly, it is clear that cuprizone intoxication did take place with the powdered cuprizone, as ASPA+ oligodendrocytes were reduced in the caudal corpus callosum with this formulation, relative to the appropriate control powdered feed. It has also been suggested that for effectively inducing demyelinating disease pathology via cuprizone, mice need to additionally intake the cuprizone powder through the skin and respiratory system^28^. However, this does not occur when cuprizone pellets are delivered. Our current study suggests this may not be as central to the pathological action of cuprizone as previously thought.

It is also important to note that mice fed control powder had significantly fewer oligodendrocytes than those fed control pellets, particularly in the rostral corpus callosum. In addition, the density of Olig2+ cells were lower in the cuprizone powder fed mice compared to their cuprizone pellet fed counterparts. This may be due to abnormal energy metabolism as mice fed powdered food are known to develop hyperglycaemia and increases in epinephrine and other catecholamines^42^. Catecholamines can be toxic to oligodendrocytes when the protective effect of astrocytes is impaired^43^, and astrocyte function is impaired under hyperglycaemic conditions^44^. This interesting finding may have implications for remyelination studies, as mice fed powdered control chow may not be suitable controls for oligodendrocyte density. In line with this notion, the loss of oligodendroglia reported in previous studies at 3 weeks with powdered cuprizone^45–48^ only became apparent when we compared powdered cuprizone to pelleted control. As these earlier studies either used pelleted feed as control for powdered cuprizone or did not specify the formulation of their control feed, the seeming discrepancy in oligodendroglia loss between such studies and ours may relate to the formulation of control feed used to assess the effect of cuprizone. These data highlight the importance of using control feed in the same formulation as the cuprizone-containing feed to evaluate the effect of cuprizone. The effect of powdered feed on oligodendroglia should also be considered when designing remyelination studies, as a change to powdered control chow from powdered cuprizone diet may not reflect a full return to a healthy mature oligodendrocyte population that is able to remyelinate fully.

Both powder and pellets produced the well-known rostro-caudal pattern of demyelination, whereby the medial caudal region of the corpus callosum is more susceptible to cuprizone insult^34,40,49,50^. Regionally specific susceptibility may be due to the proximity of the medial rostral corpus callosum to the subventricular zone, as neural precursor cells from the subventricular zone migrate into the proximal rostral corpus callosum by week 3 of cuprizone administration, where they can dampen the inflammatory response and differentiate into mature oligodendrocytes^26,51–53^. It is important to note however that despite the midline being most susceptible to demyelination in the caudal regions, in the rostral corpus callosum the lateral areas are more susceptible to cuprizone intoxication^39^. Since the lateral corpus callosum was not analysed in the current study, the effect of the cuprizone formulations on inducing demyelination in these regions may have been missed. Mice fed powdered cuprizone displayed greater within-group variability than those fed cuprizone pellets, perhaps due to variable intake of the powdered and possibly less palatable feed. The variability in certain outcomes in mice fed cuprizone powder may have been a factor in the lack of statistically significant differences compared to relative controls. Therefore, the reduced variability with mice fed cuprizone pellets is another advantage of the pelleted cuprizone technique.

When utilising animal models, it is critical that the features of the model are replicated across studies, including the time at which aspects of the pathology begin to become detectable. The most widely recognized hallmarks of cuprizone-induced toxicity are the loss of mature myelinating oligodendrocytes and demyelination. Death of oligodendrocytes occurs from around 2–7 days into cuprizone administration, and reduced oligodendrocyte density is still apparent at 3 weeks, whilst demyelination becomes detectable from around 3 weeks^10,39,54^. The demyelination observed in this study in the cuprizone pellet group is in the early phase at 3 weeks, and a longer period of exposure to cuprizone of 5 weeks may have resulted in a more severe demyelinating effect^39^. Indeed, at 5 weeks, powdered cuprizone has been found to be effective at inducing demyelinating pathology^39^. The lack of significant demyelination observed with 3 weeks administration of cuprizone powder may reflect delayed cuprizone intake, rather than a complete lack of efficacy, given the significant loss of mature ASPA+ oligodendrocytes seen in this group. The lack of change in Olig2+ and CC1+ cell densities with powdered feed may reflect the contribution to cell densities of OPCs^55^ and astrocytes^56^ respectively. The demyelination and mature oligodendrocyte depletion observed with pelleted cuprizone indicates that the potency of cuprizone was not compromised by the copper contained in standard rodent chow. Moreover, our powdered feed contained the same amount of copper as the pelleted feed, allowing reliable interpretation of the effects of feed formulation on demyelination pathology. The observed oligodendropathy and demyelination were complemented by a robust inflammatory response. It is known that by 1 week of cuprizone administration, microglia are activated in the corpus callosum^23^, with astrocytes showing morphological changes as early as 1 week and increasing in density by 3 weeks^24^. In the current study, similar inflammatory changes were accompanied by increased immunoreactivity of oxidative stress markers for protein nitration and DNA damage, which we have previously demonstrated following 3 weeks of cuprizone administration^57^. The higher protein nitration in rostral corpus callosum with powdered cuprizone compared to the caudal region is an interesting finding that warrants further mechanistic investigations. There is limited information available on the effects of cuprizone intoxication on protein nitration in the rostral and caudal corpus callosum. A plausible hypothesis is that high protein nitration is a response to cuprizone intoxication when the initial density of mature oligodendrocytes is low in the rostral region. Consistent with this hypothesis, we found that the density of mature oligodendrocytes in the rostral region was lower with control powder when compared to pelleted control. Future studies identifying the cell types and mechanisms responsible for high protein nitration in the rostral corpus callosum will allow better understanding of the regional differences with powdered and pelleted cuprizone. Given the lack of change in cell densities, the observed tissue swelling is likely to be due to increased water content and oedema, which has been found to occur as early as 3 days into cuprizone exposure and continue to at least 5 weeks^58^. The reported reparative mechanism of OPC migration to—and proliferation in—the corpus callosum, particularly caudally, at 3 weeks of cuprizone administration^25,26^, was also observed here. In sum, the pathological features we demonstrate (particularly when mice were fed cuprizone pellets) are consistent with the literature descriptions of the cuprizone-induced model of demyelinating disease when assessed at the later 5 week timepoint and beyond.

Taken together, this study found that at a 3 week timepoint, cuprizone pellets were more efficacious than cuprizone powder at producing astrocyte reactivity, microglial activation, oxidative stress, tissue swelling, and a reduction in the density of mature oligodendrocytes and demyelination when compared to control feeds, particularly in the caudal corpus callosum. These results indicate that cuprizone pellets are a more effective method of delivering cuprizone than powdered feed to induce demyelinating disease pathology at this early 3 week demyelination timepoint.

## Materials and methods

### Animals and study design

Twenty-four 8 week old male C57Bl/6J mice were obtained from the Animal Resource Centre (Murdoch, Western Australia). The mice had ad libitum access to food and water and were housed under a 12 h light/dark cycle. All procedures were approved by the Animal Ethics Committee (AEC) of The University of Western Australia (RA/3/100/1613), with reciprocal approval from the AEC of Curtin University (ARE2019-4), and were performed in accordance with the principles of the National Health and Medical Research Council of Australia. This study is reported in accordance with the ARRIVE guidelines. Animal randomisation was achieved at a cage level by allocating animals to groups in the order in which they were received from the supplier. Animals were allowed a 1 week acclimatisation period to the experimental location prior to commencing the study. 0.2% cuprizone (ChemSupply) was delivered for 3 weeks either mixed into control powdered feed (Specialty Feeds, Perth, Western Australia) or as cuprizone-containing pellets (Specialty Feeds, Perth, Western Australia). The cuprizone-containing pellets were formed using cuprizone powder from the same batch as that delivered directly to the mice, to minimise any batch or supplier variability. Animals were given equivalent amounts of cuprizone, regardless of feed formulation. Mice fed the cuprizone pellets ate approximately 3–5 g of feed each per day, however the quantity of cuprizone powder consumed could not be determined as the mice were consistently urinating, defecating and digging around in the feed. Animals not receiving cuprizone were administered either control pellets with the same nutritional composition to the cuprizone-containing pellets (except cuprizone), or the control powder that the cuprizone powder was mixed into. The control powder was nutritionally the same as the control pellets.

The animals were divided into four experimental groups depending on feed type (N = 6/group); a control pellet group, a cuprizone pellet group, a control powder group, and a cuprizone powder group. 6 mice/group was chosen as power calculation (using G*Power Version 3.1^59^) at α-level of 0.05 showed that it was sufficient for an 80% chance of detecting the published effect size of 2.02 for immunohistochemical measures of myelin and glia in the corpus callosum following cuprizone treatment^60^. Animals were housed two animals per cage and were weighed daily, and if cuprizone-fed animals weighed less than 85% of their body weight at the start of the experimental period, they were weighed twice daily and their feed was supplemented with normal powdered chow until they returned to above the 85% threshold. One mouse fed cuprizone powder underwent this intervention; however, they were returned to cuprizone powdered feed 24 h after commencing diet supplementation due to substantial weight gain during this period. The level of myelination for this particular mouse was not found to be an outlier compared to the rest of the cuprizone powder group, as it was not 1.5 interquartile ranges outside of the 1st or 3rd quartiles as per Tukey’s outlier detection model. Therefore, the data generated from this mouse was not removed from the data set and was included in all analyses. No further animals from either cuprizone feed groups required diet supplementation due to weight loss. Cuprizone powdered feed was changed daily^39^, whilst pellets were changed every 2–3 days^34^, as per standard protocols.

### Tissue processing

Following the 3 weeks of cuprizone treatment, mice were euthanised using pentobarbitone sodium (160 mg/kg, Delvet), and transcardially perfused with 0.9% saline, followed by 4% paraformaldehyde (Sigma-Aldrich) in 0.1 M phosphate buffered solution pH 7.4. The brains were dissected and fixed overnight in 4% paraformaldehyde. The brains were transferred into 15% sucrose (ChemSupply) 0.1% sodium azide (Sigma-Aldrich) for cryoprotection and storage at 4 °C. Brains were cut down the midline into two hemispheres and block cut to isolate the region of the cortex that contained the corpus callosum. The right hemisphere was then embedded in a mould of optimal cutting temperature compound (OCT, Scigen) and fast frozen in isopentane in a bath of liquid nitrogen until 2 min after the OCT had turned opaque. Tissue was then cryosectioned coronally at − 20 °C into 24 well plates (ThermoFisher Scientific) containing PBS 0.1% sodium azide at 25 μm thickness and stored at 4 °C until use.

### Immunohistochemistry

Immunohistochemistry was performed within 24-well trays (ThermoFisher Scientific). Sections were washed with PBS three times, before being blocked in phosphate buffered saline (PBS) + 0.2% Triton X100 with 5% normal donkey serum (NDS) for 1 h at room temperature on a shaker at 40 revolutions per minute (rpm). When a primary antibody was to be used that was raised in mouse, an unconjugated donkey anti-mouse IgG (2 µg/ml, 1:1000, Abcam, Ab6707) was also included in the blocking solution. Following another wash with PBS, sections were incubated with primary antibodies recognising: activated resident microglia/macrophages (IBA1, 2 µg/ml, 1:500, Abcam, goat Ab5076); reactive astrocyte marker glial fibrillary acid protein (GFAP, 0.4 µg/ml, 1:500, Abcam, rabbit Ab33922); oxidative stress markers 3-nitrotyrosine (3-NT, 2 µg/ml, 1:500, Abcam, mouse Ab61392) and 8-hydroxy-2′-deoxyguanosine (8OHDG, concentration undetermined by manufacturer, 1:250, Novus Biologicals, goat NB600-1508); and oligodendroglial indicators oligodendrocyte transcription factor 2 (Olig2, 0.08 µg/ml, 1:250, R&D Systems, goat AF2418), platelet-derived growth factor receptor alpha (PDGFRα, 5 µg/ml, 1:200, ThermoFisher Scientific, rabbit PA5-16571), aspartoacylase (ASPA, 2 µg/ml, 1:500, EMD Millipore, rabbit ABN1698), and adenomatous polyposis coli (APC) clone (CC1, 0.2 µg/ml, 1:500, ThermoFisher Scientific, mouse MA1-25884). Primary antibodies were diluted in PBS + 0.2% Triton X100 with 5% NDS. Following a 30 min incubation with primary antibodies at room temperature on a shaker at 40 rpm, the well plates were transferred to 4 °C for overnight incubation on a shaker at 40 rpm. Sections were then washed three times before being incubated with secondary antibodies (1:400, species-specific Alexa Flour 488, 555 or 647, Thermo Fisher Scientific), diluted in PBS for 2 h at room temperature on a shaker at 40 rpm. Finally, sections were washed another three times before being incubated with Hoechst 3342 (1:2000, Thermo Fisher Scientific) and washed another three times. Sections were then mounted onto slides using fine paintbrushes, and coverslipped with Fluoromount-G (Thermo Fisher Scientific).

### Histology

Myelin Black-Gold II (Biosensis) was performed within 24-well plates (ThermoFisher Scientific) in line with the manufacturer’s instructions. Staining of all sections was conducted in a single session. In brief, sections were washed with distilled water before being immersed in 1 × Black-Gold II solution and incubated at 65 °C for 14 min, until staining of the myelinated regions was becoming apparent. Following three washes with distilled water, sections were mounted onto slides and imaged within the same day.

### Imaging and analysis

For immunohistochemical outcome measures, the rostral and caudal medial corpus callosum were visualised using a Nikon A1 confocal microscope (Nikon Corporation, Australia, Coherent Scientific), and a series of 13 images were taken at 0.5 μm increments along the z-axis at a magnification of 20 × and a numerical aperture of 0.75. For histological Black-Gold II, the rostral and caudal corpus callosum were visualised at a single visual slice along the z-axis using a Nikon Eclipse Ts2 microscope (Nikon Corporation, Australia, Coherent Scientific), at a magnification of 20 × and a numerical aperture of 0.25. The rostral corpus callosum was imaged at approximately 0.97 mm anterior to bregma and the caudal corpus callosum at approximately 2.15 mm posterior to bregma. Imaging for each outcome measure for each region of interest was performed in a single session with consistent microscope capture settings for all experimental groups.

The area of immunoreactivity of IBA1, GFAP, 3-NT, and 8OHDG were semi-quantified using Fiji/ImageJ software. In brief, a single visual slice was selected, and the total area of the corpus callosum was quantified. A threshold was set to distinguish positive immunofluorescence from background, and an intensity analysis was performed to determine the area of intensity above threshold within the region. The data were then normalised against the total area of the corpus callosum analysed. The area of the corpus callosum in a 20 × image was also quantified using Fiji/ImageJ, by determining the area of the region of interest within the total image, based on Hoechst+ nuclei.

Hoechst+, Olig2+, PDGFRα+, Olig2+/PDGFRα+, ASPA+, and CC1+ cells were quantified using the ZEISS Zen Intellesis image analysis software, which uses deep learning to automatically identify cells of interest based on a range of features from the image, including pixel intensity and cell morphology^61^. Data are expressed per mm^2^ to provide a cell density for the region of interest.

For Black-Gold II, analysis was performed similar to as previously described^62^. In brief, using Fiji/ImageJ software, the raw image was split into three channels as an RGB stack, with the green channel selected for analysis due to its high contrast. The corpus callosum was selected and the total area measured. A threshold was set to distinguish the myelin from demyelinated regions/grey matter, and the area of myelin positive pixels was quantified and normalised against the total area of the corpus callosum, determining the percentage myelination present.

Blinding to animal identity was not possible as investigators needed to evaluate the range of immunoreactivity across groups to determine intensity thresholds. Analyses were subsequently performed objectively using automated algorithms in the ZEISS Zen Intellesis and Fiji/ImageJ.

### Statistical analyses

Graphpad PRISM 9 software was used for data analysis and illustration. A three-way repeated-measures ANOVA was used to analyse changes in animal weight over time. Sphericity was assessed using the Greenhouse–Geisser epsilon value in conjunction with the Greenhouse–Geisser adjustment, and sphericity was not assumed (ε < 0.75). To detect changes between groups within timepoints, two-way ANOVAs were performed at each timepoint using Tukey’s post-hoc test. For all other outcomes, two-way ANOVA with Tukey’s post-hoc test was performed across all four groups. Statistical significances shown on graphs are hypothesis-driven and may not display all significant differences obtained. Specifically, only significant differences between the respective control and cuprizone groups are shown, as well as differences between the two cuprizone formulations and the two control feed formulations. Outliers were not removed for any of the outcome measures. p values in the text are for post hoc comparisons.

## Data Availability

The datasets generated during and/or analysed during the current study are available from the corresponding author on reasonable request.
